# Insight into the potential significance of miR-760 and miR-1973 in breast cancer: a comprehensive analysis

**DOI:** 10.1038/s41598-026-44175-3

**Published:** 2026-04-01

**Authors:** Sara A. Kamal, Walid E. Zahran, Reham A. A. Elshimy, Doaa M. Ibrahim

**Affiliations:** 1 Biochemistry Department, Faculty of Science, Ain Shams University, Cairo, Egypt; 2 Department of Clinical and Chemical Pathology, National Cancer Institute, Cairo University, Cairo, Egypt

**Keywords:** miR-760, miR-1973, CA 15.3, Breast cancer, Bioinformatic analysis, Biomarkers, Cancer, Computational biology and bioinformatics, Genetics, Oncology

## Abstract

Aberrant expression levels of miR-760 and miR-1973 were observed in different solid tumors; however, their expression levels in breast cancer (BC) remain unclear. This study aimed to evaluate their expression levels, assess their relationship with the clinicopathological features, and explore their molecular mechanisms using bioinformatic analysis. MiRNAs’ expression levels and CA 15.3 concentration were measured in 130 patients and 30 healthy controls. Databases were used to predict their target genes, functional enrichment analyses were carried out, and PPI network was constructed. Additionally, the Kaplan-Meier (KM) plotter online database was utilized for survival analysis. Both miRNAs’ levels were significantly overexpressed in BC patients and more pronounced in the early stage. Further, the diagnostic abilities of miR-760 and miR-1973 were superior in discriminating BC patients than CA 15.3. These miRNAs were associated with increased susceptibility to BC. Based on bioinformatic analysis, 4 hub genes were identified and implicated in significant signaling pathways in BC. KM plotter demonstrated that BC patients with overexpressed miR-760 had shorter overall survival. In conclusion, this study provides novel insights into the clinical utility of miR-760 and miR-1973 as non-invasive biomarkers for BC and sheds light on their important contribution to BC pathogenesis.

**Supplementary Information:**

The online version contains supplementary material available at 10.1038/s41598-026-44175-3.

## Introduction

Worldwide, breast cancer (BC) is the most common cancer and a leading cause of cancer-related death in women. BC has been classified into three subtypes: luminal-like BC that expresses high levels of the estrogen (ER) and progesterone receptors (PR) but not human epidermal growth factor receptor 2 (HER2); the second subtype shows a high expression of HER2 and low expression of both ER and PR. The last one is triple-negative because this subtype expresses neither ER, PR, nor HER2^1^. The early diagnosis of BC and monitoring of the disease progression result in improved response to treatment and increased survival rates. Although imaging techniques such as mammograms, MRI, CT, and PET are the most used diagnostic tools, the cost incurred and limited sensitivity have hampered their use^2^. Accordingly, in an alternative approach, other serum biomarkers such as carcinoembryonic (CEA) and carbohydrate antigens (CA 15.3) were widely employed but have many inherent deficiencies. Moreover, these biomarkers are only applied to monitor disease recurrence, not for the early diagnosis^3^. Therefore, there is a pressing need to explore non-invasive biomarkers for the early detection of BC that might remedy the defects of imaging examinations and tumor markers.

MicroRNAs (miRNAs) are a class of short non-coding RNAs of 19–25 nucleotides that regulate gene expression post-transcriptionally *via* direct interaction and degradation of target mRNAs^4,5^. Numerous studies have indicated that aberrant expression of miRNAs is associated with various cancers and exhibits a vital role in improving the diagnosis and prognosis of cancer^6–8^. As reported, miRNAs can regulate several biological processes like cell differentiation, proliferation, apoptosis, and migration, which are involved in cancer aggravation^9,10^.

MiRNAs could act as tumor suppressors or onco-miRs in BC according to their target genes^11,12^. Further, deregulated expression of several miRNAs was reported to be associated with shorter overall survival (OS) and/or worse disease-free survival (DFS), including miR-10b, miR-21, and miR-205^13–17^.

MiR-760 is located on chromosome 1 at position 1p22.1 in the intronic − 1 region of the *BCAR3* gene and can be regulated by estrogen^18^. Even though many studies on different cancer types have revealed that miR-760 expression was involved in disease diagnosis and progression, data about its levels were contradictory. A low expression level of miR-760 was found in colorectal, gastric, and non-small cell lung cancer^19–21^. Otherwise, an elevation in miR-760 level was observed in ovarian cancer^22^. Regarding BC, few and limited studies were focused on miR-760 role and prognostic efficacy in cancer progression,^23–25^ however, its circulating levels and relation with clinicopathological features are still unclear.

Despite little evidence available on the differential expression of miR-1973 in cancer, researchers displayed deregulated expression levels of miR-1973 in many solid tumors such as prostate cancer, ovarian cancer, renal cell carcinoma, and colon carcinoma, providing an oncogenic potential of this miRNA.^26–29^ Further, only one study by Fomicheva et al. evaluated miR-1973 expression level in the breast cancer MDA-MB-231 cell line^30^.

Because of this variability and few studies performed mainly on cell lines or tissues, this study was designed to evaluate circulating miR-760 and miR-1973 levels in BC and to explore their clinical utility as potential diagnostic biomarkers. Moreover, the prognostic potential and biological functions of these miRNAs in BC were explored using bioinformatic analyses.

## Subjects and methods

### Subjects

In total, 160 participants were enrolled in this study and divided into 30 patients with benign breast hyperplasia (BBH) and 100 patients with newly diagnosed BC who had not received any treatment strategies. In addition, 30 age-matched females with no history of cancer and no inflammatory conditions served as a healthy control group. Patients who took any treatment, underwent chemo-or radiotherapy, or had any other disease or type of cancer were excluded. Patients were consecutively enrolled from the Medical Oncology department at the National Cancer Institute (NCI), Cairo University, Cairo, Egypt. This study was performed in accordance with the Declaration of Helsinki guidelines and all subjects signed informed written consent. Ethics Committee and Institutional Review Board of National Cancer Institute, Cairo University, Cairo, Egypt, has approved this study (IRB approval code# 202101-04-02001). The diagnosis of BC and benign breast lesions were confirmed by histopathological examination of breast biopsy specimens and imaging techniques according to the World Health Organization categories. To determine molecular subtypes, expression of ER, PR, and HER2 was evaluated using immunohistochemistry. The tumor stage was assessed according to the American Joint Committee on Cancer tumor-node-metastasis (TNM) classification. In addition, clinical data was collected from the patient’s files.

### Methods

5 mL of venous blood samples were obtained from all participants, left to clot, and centrifuged at 2000 ×g for 10 min. The separated sera were aliquoted into microcentrifuge tubes and stored at -80 °C till the analysis.

CA 15.3 level was determined in serum samples using an ELISA kit and following the manufacturer’s instructions (Cat# 10104, Chemux Bioscience Inc., USA).

#### MicroRNA extraction, reverse transcription, and real-time quantitative PCR

MiRNeasy Mini Kit (Cat# 217004, Qiagen, Germany) was used according to the manufacturer’s instructions to extract total RNA including miRNAs from serum samples. Purified RNA concentrations and quality were measured using a NanoDrop spectrophotometer (Thermo Scientific) and stored at -80 °C.

RNA was reverse transcribed using TaqMan™ MicroRNA Reverse Transcription kit (Cat# 4366596, Thermo Fisher, DE, USA). cDNA synthesis was performed in a total volume of 15 µL according to the kit instruction.

Q-PCR was conducted utilizing TaqMan™ Gene Expression Master Mix (Cat# 4370048, Applied Biosystems) and TaqMan™ miRNA qPCR Assays (Cat# 4427975, Applied Biosystems) for miR-1973, miR-760, and miR-484 (Assay ID: 245468, 002328, and 001821 respectively). MiR-484 was used as an internal control based on several studies, which reported that its expression level was stable between the healthy subjects and BC patients^31,32^. A PCR reaction of 20 µl final volume contained 10 µl of 2× TaqMan^®^ Universal PCR Master Mix, 1 µl of cDNA as a template, 1 µl of 20× TaqMan™ MicroRNA Assay then it was completed with nuclease-free water. qPCR reactions were carried out using Step One™ Real-Time PCR System (Applied Biosystems, CA, USA) as follow: 50 °C for 2 min, 95 °C for 10 min, 45 cycles of 95 °C for 15 s, and 60 °C for 1.15 min. Each analysis was done in triplicate and the 2^−ΔΔCt^ method was used for relative expression calculations^33^.

#### Overall survival analysis of miR-760 and miR-1973 in BC Patients

Kaplan-Meier (KM) plotter database (https://kmplot.com/analysis/index.php?p=service), an integrated online bioinformatics tool, was used to assess the prognostic significance of both miRNAs in BC patients. Among datasets included in the KM plotter, the Molecular Taxonomy of Breast Cancer International Consortium (METABRIC) cohort dataset was adopted to assess the association between the two studied miRNAs and OS in BC patients. The dataset includes data from 1262 patients with long follow-up period (median: 94 months) and average characteristics (78% ER-positive and 12% HER2-positive)^34^. However, the analysis was restricted to systemically untreated patients (*n* = 199) to be consistent with our patient cohort. The patients were split into two groups according to the auto select best cutoff feature of each proposed miRNA.

#### Collection of miR-760 and miR-1973 target genes and BC-related genes

Several online databases, including miRWalk v3.0 (http://mirwalk.umm.uni-heidelberg.de/) with a binding probability ≥ 0.95, TargetScanHuman Release 8 (https://www.targetscan.org/vert_80/), miRTarBase (https://mirtarbase.cuhk.edu.cn), and DIANA-microT 2023 (https://dianalab.e-ce.uth.gr/microt_webserver/#/) were used to predict miR-760 and miR-1973 target genes at the 3′ untranslated region (3′UTR)^35–37^.

Further, breast cancer and breast carcinoma were entered as keywords into the GeneCards (https://www.genecards.org) and DisGeNET (https://www.disgenet.org/) to identify BC-related targets^38,39^.

The interaction between the miRNAs’ targets and the BC-related genes was presented by a Venn diagram to acquire the common genes.

#### GO and pathway enrichment analyses

The database for annotation, visualization, and integrated discovery (DAVID; Version 6.8) (https://david.ncifcrf.gov/), was used to carry out gene ontology (GO) functional enrichment analysis and Kyoto encyclopedia of genes and genomes (KEGG) pathway enrichment analysis on the intersected genes^40,41^. The GO terms were categorized into three types: Biological process (BP), cellular component (CC), and molecular function (MF). FDR-adjusted *p* < 0.05 was considered statistically significant.

#### Construction of protein-protein interaction

A protein-protein interaction (PPI) network was constructed using the search tool for the retrieval of interacting genes (STRING; v12, http://string-db.org/) to explore the interaction among the obtained common genes. The selected target proteins were limited to *Homo sapiens*, and a minimum interaction score was set to medium confidence (0.400).

Subsequently, the PPI was visualized by Cytoscape software (v3.10.1, www.cytoscape.org/), and the topological properties of network nodes were analyzed using the Cytoscape Network Analyzer tool. The node size and color were set proportional to the node connectivity degree to facilitate the visual identification of the network. Further, the molecular complex detection (MCODE; v2.0.3, http://apps.cytoscape.org/apps/mcode) plug-in was used to select the biologically relevant subsets of network-related genes from the entire set. MCODE defines the seed node as the node with the highest weighted vertex.

#### Statistical analysis

SPSS version 20.0 (IBM Corp., NY, US) was employed for data analysis. For numerical data, the Shapiro–Wilk test was used to determine the type of data distribution. Normally distributed data were described as mean ± standard deviation, while median and interquartile range (25th and 75th percentile) were used to display the non-normally distributed data. Categorical data were depicted as frequencies and percentages. In addition, TNM stages (I and II) were considered an early stage. Mann-Whitney *U* test was employed to compare the non-normally distributed data between two groups. The difference between groups was compared using one-way ANOVA test followed by Tukey’s post hoc or Kruskal Wallis test followed by Dunn test as appropriate. Where Chi- square analysis was carried out for categorical variables.

To assess the discriminative capacity of serum miR-760 and miR-1973 levels for BC patients, receiver operating characteristic (ROC) curve analysis was carried out, and the optimal cutoff values were determined using the Youden index J. To assess the strength of the association between serum levels of miR-760 and miR-1973 and the susceptibility to BC, logistic regression analysis was performed using the expression as an independent continuous variable. The strength of the association was adjusted for age, the presence of family history, and menstruation as potential confounders. All *p*-values ≤ 0.05 were considered significant.

## Results

### General characteristics of study population

Table 1 presents clinical and laboratory features of the studied groups. There was no significant difference in age and menstruation state among all groups. Majority of BC patients were grade II (72%) and had positive estrogen and progesterone receptors (72% and 75%, respectively). In comparison with the control group, serum CA 15.3 was significantly elevated in BC patients (*p* < 0.001). Additionally, the expression levels of miR-760 and miR-1973 were significantly up-regulated in BC patients by 51.6% and 358% (*p* = 0.038 and *p* < 0.001, respectively). Meanwhile, patients with BBH exhibited a borderline significance in serum level of CA 15.3 (*p* = 0.06) and displayed a non-significant elevation in either miR-760 or miR-1973 expression levels (*p* = 0.556 and *p* = 1, respectively).

**Table 1:** Clinical, laboratory characteristics and miRNAs expression levels in the study population.

Variables	Control (*n* = 30)	BBH (*n* = 30)	BC (*n* = 100)	*p*-value
Age (years)	46.23 ± 9.14	44.57 ± 15.11	48.02 ± 9.60	0.277
Family history, n (%)				
Yes	0 (0)	16 (53.3)	28 (28)	
No	30 (100)	14 (46.7)	72 (72)	
Menstruation, n (%)				
Pre- menopause	23 (76.7)	21 (70)	68 (68)	0.662
Post- menopause	7 (23.3)	9 (30)	32 (32)	
Histological grade, n (%)				
I	-	-	6 (6)	
II			72 (72)	
III			22 (22)	
Stage, n (%)				
I	-	-	18 (18)	
II			26 (26)	
III			36 (36)	
IV			20 (20)	
Lymph nodes, n (%)				
Positive	-	-	84 (84)	
Negative			16 (16)	
ER status, n (%)				
Positive	-	-	72 (72)	
Negative			28 (28)	
PR status, n (%)				
Positive	-	-	75 (75)	
Negative			25 (25)	
HER-2 status, n (%)				
Positive	-	-	31 (31)	
Negative			69 (69)	
Molecular subtype, n (%)				
Luminal A	-	-	34 (34)	
Luminal B			35 (35)	
HER-2 Enriched			23 (23)	
Triple negative			8 (8)	
CA15-3 (U/mL)	8.84 (6.80-13.06)	11.98 (7.87–19.89)	14.61 (9.78- 27)^a^	< 0.001
miR-760 relative expression	1.59 (1.59–2.10)	1.29 (0.35–2.25)	2.41 (1.12–3.40)^a, b^	< 0.001
miR-1973 relative expression	0.84 (0.22–4.11)	1.45 (1.03–6.68)	3.85 (1.45–8.23)^a, b^	< 0.001

Normally distributed variables are expressed as mean ± SD, non-normally distributed variables as median (inter-quartile range), and categorical variables as frequencies (percentages). In multiple comparisons, ^a^*p*<0.05 vs. normal control group, ^b^*p*<0.05 vs. benign group. ER: estrogen receptor; PR: progesterone receptor; HER-2: human epidermal growth factor receptor-2.

Compared to the BBH group, BC patients exhibited a significant elevation in the expression levels of miR-760 and miR-1973 by 86.8% and 165% (*p* < 0.001 and *p* = 0.001, respectively).

BC patients with an early stage displayed a significant elevation in the expression levels of both miR-760 and miR-1973 compared to the controls (benign and healthy control groups) with a median and interquartile range of [1.95 (1.12–3.17) vs. 1.58 (1.12–2.09), *p* = 0.025 and 6.68 (1.45–8.22) vs. 1.1 (0.55–4.11), *p* < 0.001, respectively]. Whereas serum CA 15.3 levels showed a non-significant difference between patients with an early stage and controls [13.38 (9.02-18) vs. 10.04 (7.07–15.03), (*p* = 0.111)].

#### Association of miR-760 and miR-1973 with clinicopathological features in BC patients

Based on the median miRNA expression levels, the BC patients were assigned into two groups (high and low). Family history, menstruation state, lymph node involvement, and receptor status, did not differ significantly between low and high miR-760 expression groups.

Supplementary Table 1 showed a significant association between the high miR-760 expression level and the late stage of the disease (*p* = 0.002). Meanwhile, the association with molecular subtypes did not survive after applying Bonferroni correction for multiple hypotheses (*p* = 0.021).

Regarding miR-1973, no significant associations were found with family history, histological grade, stage, lymph node involvement, receptors status, and molecular subtypes. Notably, the association between the serum expression level of miR-1973 and the menstruation state did not survive after applying Bonferroni correction for multiple hypotheses (*p* = 0.010), as shown in Supplementary Table 2.

#### Diagnostic efficacy of miR-760 and miR-1973 expression levels for BC patients

**Fig. 1:**
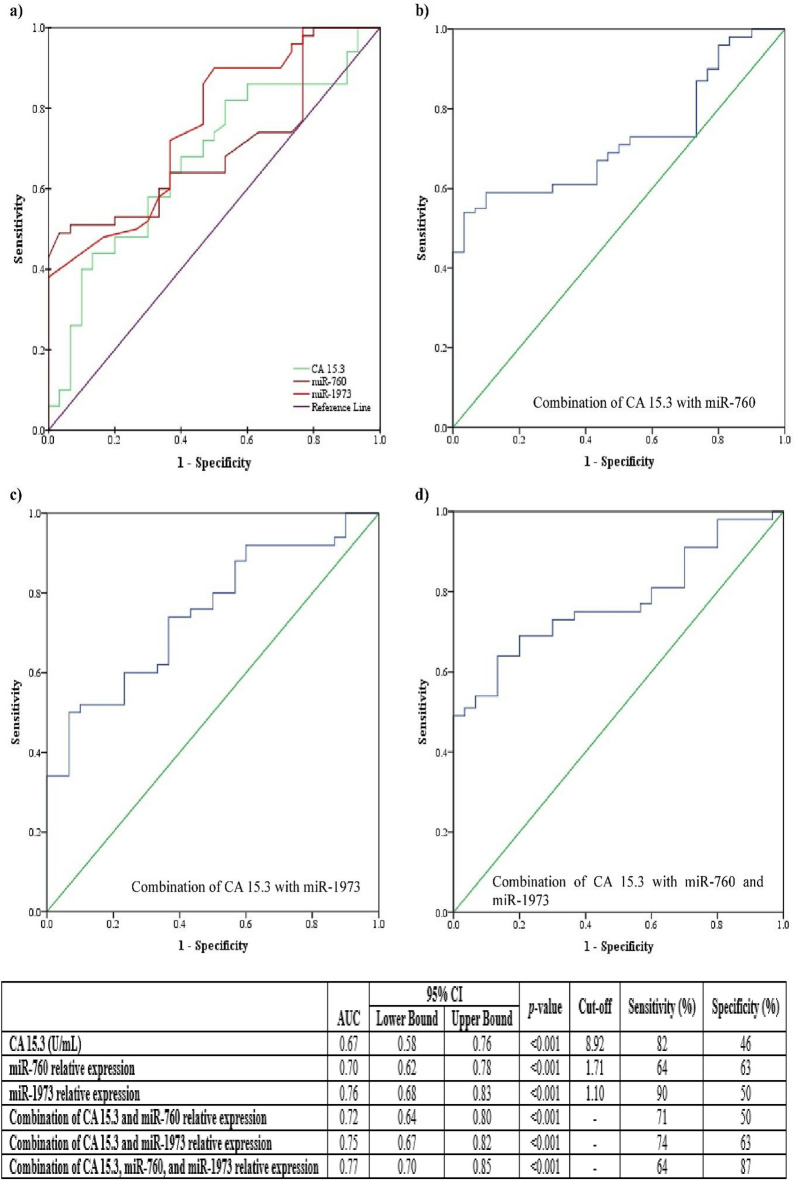
ROC curves of (**a**) miR-760, miR-1973 relative expression and CA15.3 concentration; (**b**-**d**) their combinations with CA15.3 to discriminate breast cancer patients from the control and benign breast hyperplasia groups. AUC: area under curve, CI: Confidence interval.

Figure 1 demonstrates the ROC curves of serum miR-760, miR-1973 expression levels, CA 15.3 concentration, and their combinations to discriminate BC patients from the control and BBH groups. The figure shows that CA 15.3 had the lowest diagnostic value for BC with an area under curve (AUC) of 0.67 (95% confidence interval [CI]: 0.58–0.76, *p* < 0.001) at an optimal cutoff point of 8.92 U/mL, which could yield 82% sensitivity and 46% specificity. Meanwhile, both miR-760 and miR-1973 showed better diagnostic values. MiR-760 had 64% sensitivity, and 63% specificity with an AUC of 0.70 (95%CI: 0.62–0.78, (*p* < 0.001) and a cut-off point of 1.7. Otherwise, the AUC of miR-1973 was 0.76 (95%CI: 0.68–0.83, *p* < 0.001) at an optimal cut-off point of 1.1 that is associated with sensitivity and specificity of 90% and 50%, respectively.

Notably, five-fold cross-validation was performed to evaluate the discriminative performance of CA 15.3, miR-760, and miR-1973. The mean cross-validated AUC was 0.67, 0.71, and 0.75, respectively.

Concerning the combination of miRNAs with CA 15.3, the combinational ROC analysis illustrates that the combination of CA 15.3 with miR-760 and miR-1973 increased sensitivity and/or specificity values. Whereas the combination of CA 15.3 and miR-760 resulted in sensitivity and specificity of 71% and 50%, respectively, with an AUC of 0.72, the combination of CA 15.3 with miR-1973 showed an AUC of 0.75, with 74% sensitivity and 63% specificity. Nevertheless, the best diagnostic ability was obtained by combining the three parameters, with an AUC of 0.77, 64% sensitivity, and 87% specificity.

#### MiR-760 and miR-1973 as risk factors for BC

Table 2 shows the results of the binary logistic regression analyses performed to test the associations of miR-760 and miR-1973 serum levels with the risk of developing BC. MiR-760 and miR-1973 were associated with an increased risk of BC development by 200% and 130% respectively. This result remained significant after the adjustment of the potential confounders such as age, family history, and menstruation state.

**Table 2:** Binary logistic regression analysis of miR-760 and miR-1973 as risk factors of breast cancer.

	Crude OR (95% CI)	*p*-value	^†^Adjusted OR (95% CI)	*p*-value
Age	1.02 (0.99–1.1)	0.137		
Family history	1.1 (0.52–2.19)	0.855		
Menstruation	1.29 (0.64–2.63)	0.477		
miR-760 relative expression	1.97 (1.43–2.72)	< 0.001	2.15 (1.51–3.07)	< 0.001
miR-1973 relative expression	1.29 (1.14–1.46)	< 0.001	1.34 (1.17–1.52)	< 0.001

OR: Odds ratio, 95% CI: 95% confidence interval, ^†^: adjusted for age, family history, and menstruation as potential confounders.

The appropriate sample size and power calculations for regression analysis were carried out using the G*Power software version 3.1.9.2 (Düsseldorf University, Germany). At the level of α error probability = 0.05, calculations showed that the sample size can give as high as 91.5% and > 95% power for the miR-760 and miR-1973 expression levels, respectively.

#### Association between the miRNAs and OS in BC patients

**Fig. 2:**
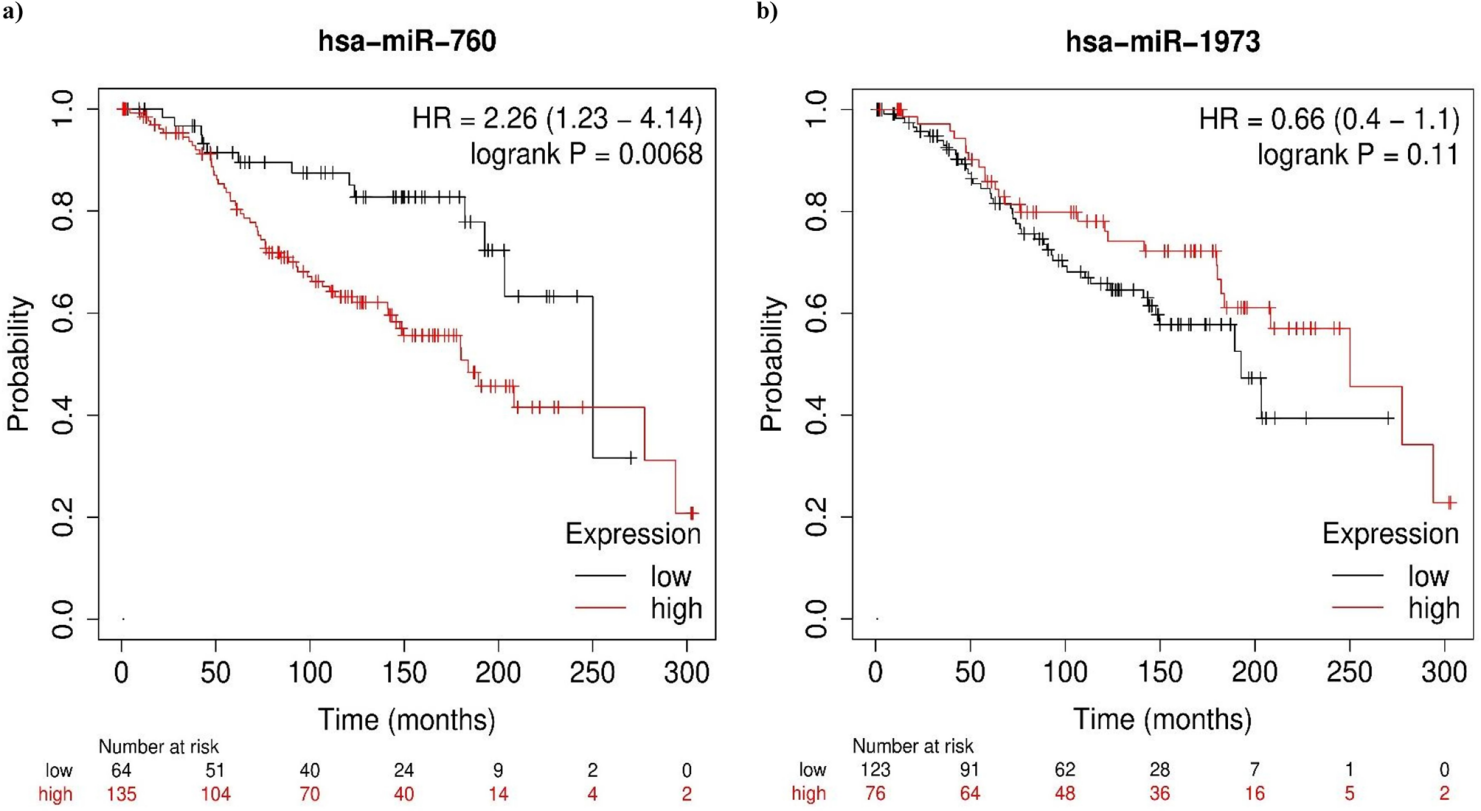
Overall survival curves for (**a**) miR-760 and (**b**) miR-1973 in 199 BC patients using the KM plotter miRNA breast cancer online database.

Next, the prognostic utility of both miRNAs expression level was examined in BC using the Kaplan-Meier (KM) plotter database (Fig. 2). The OS was significantly lower in patients with high expression of miR-760 compared to those with low expression (Median survival: 183.85 vs. 250.06 months, HR = 2.26, 95%CI = 1.23–4.14, *p* = 0.0068). Meanwhile, no statistically significant difference was observed in the OS rate between BC patients with high and low expression of miR-1973 (Median survival: 250.06 vs. 192.66 months HR = 0.66, 95%CI = 0.4–1.1, *p* = 0.11). Hence, the KM plotter results emphasized the prognostic significance of miR-760 in BC.

#### MiRNAs target identification

**Fig. 3:**
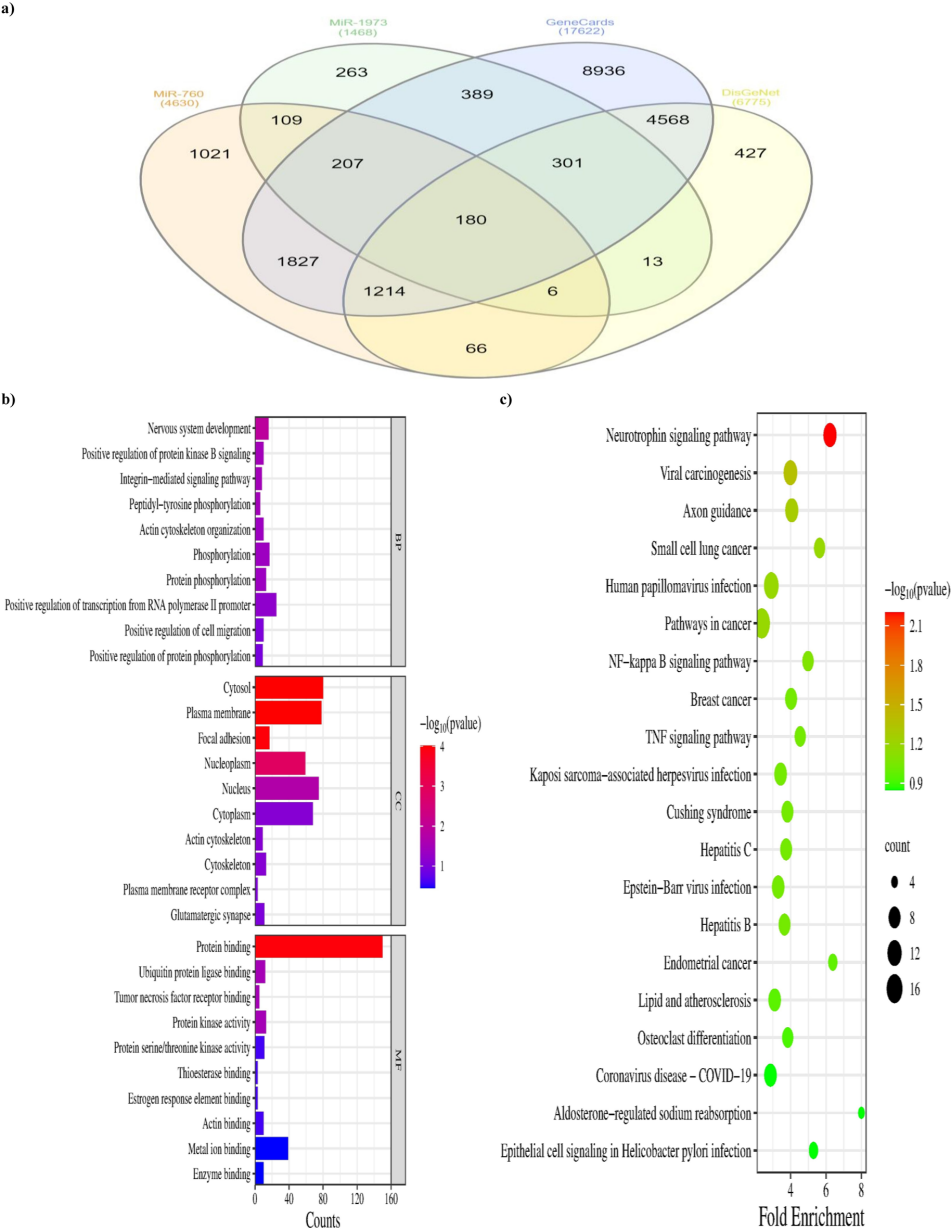
Identification of miR-760 and miR-1973 potential targets in breast cancer and enrichment analyses. (**a**) Venn diagram of the miRNAs’ targets, and breast cancer-related genes. (**b**) Bar chart of GO terms analysis showing the top 10 terms in each of biological process (BP), cellular component (CC), and molecular function (MF). The color of the bars denotes the FDR-adjusted *p*-value, and the length represents the number of genes enriched in the term. (**c**) A Bubble chart of the KEGG pathway enrichment analysis showing the top 20 putative signaling pathways according to the FDR-adjusted *p*-value^41^. The color of the circles represents the *p*-value, and the size represents the number of genes enriched in the pathway. The direction towards red color indicates more significance.

After merging and removing duplicates, mirwalk v3.0, TargetScanHuman Release 8, miRTarBase, and DIANA-microT 2023 databases predicted that miR-760 and miR-1973 target 4630 and 1468 genes respectively. Additionally, 17,622 and 6775 BC-related genes were identified from the GeneCards and DisGeNET databases, respectively (Supplementary Files 1, 2, and 3). The targets were then mapped *via* a Venn diagram, yielding 180 intersected genes that were considered, in turn, potential targets of miR-760 and miR-1973 in BC (Fig. 3a).

#### Functional enrichment analysis

GO and KEGG enrichment analyses were conducted on the intersecting targets to clarify the fundamental mechanism of the miRNAs. Of 131 BP, 48 CC, and 45 MF terms identified, only 7, 5, and 4 were statistically significant, respectively (Supplementary File 4). According to the *p*-value, the top 5 terms were nervous system development, positive regulation of protein kinase B signaling, integrin-mediated signaling pathway, peptidyl-tyrosine phosphorylation, and actin cytoskeleton organization in the BP category. The strongly connected MFs with these biological processes were protein binding, ubiquitin protein ligase binding, tumor necrosis factor receptor binding, and protein kinase activity. Further, these processes occurred mainly in cytosol, plasma membrane, focal adhesion, nucleoplasm, and nucleus. The top 10 enriched terms in each category according to the *p*-value were selected for visualization (Fig. 3b).

Moreover, the results of KEGG enrichment analysis demonstrated that these targets are enriched in 61 pathways, of which 2 pathways are statistically significant, mainly involving neurotrophin signaling pathway and viral carcinogenesis. The top 20 pathways according to the *p*-value are represented in Fig. 3c.

#### PPI network analysis

**Fig. 4:**
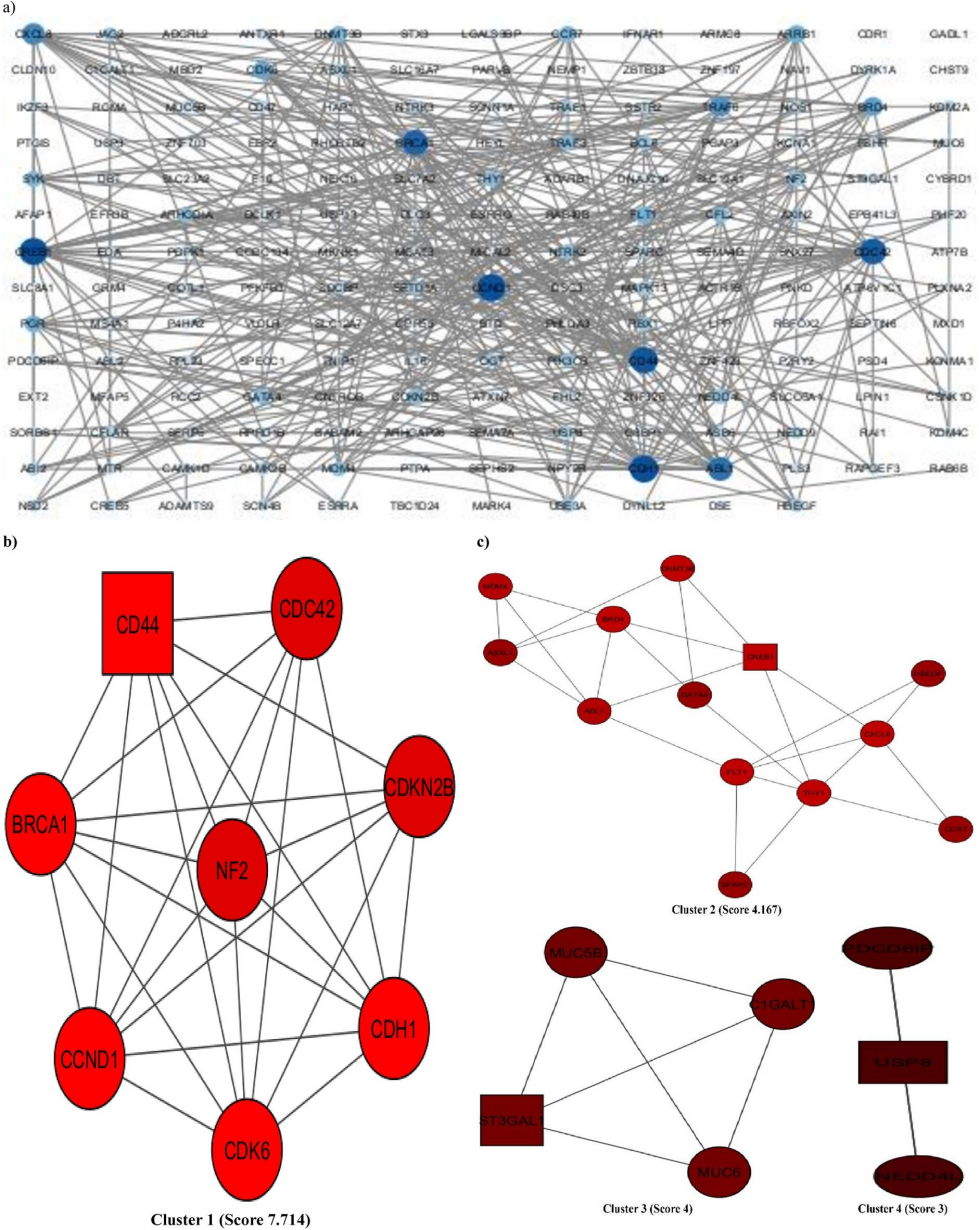
PPI network analysis. (**a**) STRING database was used to analyze the potential targets, giving a network composed of 180 nodes and 379 edges, which was then displayed on Cytoscape. The node size and color reflect the node degree, and the edges represent the interactions between nodes. (**b**) and (**c**) PPI network based on clustery analysis using the MCODE plug-in produced 4 modules, the top with a score of 7.714, 8 nodes, and 27 edges. The node color is according to the MCODE score, and the node shape is according to the node status; circles represent clustered nodes, and the rectangle represents the seed node.

The PPI network was constructed based on the 180 putative targets of the miRNAs in BC. The network visualization and analysis were performed using Cytoscape software. As presented in Fig. 4a, the network contains 180 nodes and 379 edges with an average node degree of 4.21, an average local clustering coefficient of 0.38, and a PPI enrichment p-value = 2.72e-13. The characteristic path length between all node pairs was 3.19, whereas the network diameter, radius, density, and heterogeneity were 8, 5, 0.04, and 1.068, respectively. The topological features of each node, including but not limited to Degree, Betweenness centrality, and Closeness centrality, were analyzed by the Cytoscape Network analyzer tool (Supplementary File 5). The node size and color in the network were positively related to the node degree. When the targets were sorted by degree, *CREB1*, *CCND1*, *CDH1*, *CD44*, *CDC42*, *BRCA1*, *ABL1*, *CXCL8*, *TRAF6*, and *BRD4* were identified as the 10 core targets.

Moreover, the network was further assessed with the MCODE plug-in to detect modules/clusters (densely connected regions), suggesting functional protein complexes. It revealed 4 functional modules of clustered genes sorted by their score (Fig. 4b and c). The cluster with the highest score (7.714) comprised 8 nodes and 72 edges. *CD44*, *BRCA1*, *CCND1*, *CDK6*, and *CDH1* have the highest and the same score among these genes.

Because of their high degree and MCODE score, *BRCA1*, *CDH1*, *CCND1*, and *CD44* seem to be the most important targets of miR-760 and − 1973 in BC.

## Discussion

Circulating miRNAs are considered promising biomarkers for cancer detection; however, many may also be implicated in cancer development. When identified early, 70–80% of BC patients can be cured. On the contrary, advanced BC with distant organ metastases is incurable with the currently available treatment strategies^42^. Therefore, the early detection of BC may have a crucial role in promoting disease progression and improving the survival rate. Consequently, systematic studies are needed to elucidate the predictive ability and therapeutic targets of circulating miRNAs in BC. Accordingly, this study was designed to evaluate serum expression levels of miR-760 and miR-1973, in addition to exploring their clinical utility as potential non-invasive diagnostic biomarkers.

As noticed in this study, serum miR-760 was over-expressed in early-stage BC patients compared to benign and control groups and associated with the severity of the BC stages. Consistent with our results, Huang et al.^43^ reported that miR-760 was over-expressed in the exosomes of peripheral blood and tissues of BC patients, which confirms its pivotal role in progression and metastasis of the disease. In a meta-analysis study performed by Guo et al.^23^, miR-760 levels were up-regulated in human BC tissues, suggesting that it may be one of the critical regulators associated with increased risk of BC. Further, Cicatiello et al.^44^ showed that miR-760 levels were elevated in response to estrogen in luminal MCF-7 cancer cell lines, which assumes its potential role in luminal BC. Worth mentioning, Liao et al.^22^ reported an upregulation of miR-760 in ovarian cancer, suggesting its oncogenic nature. The authors found that miR-760 targeted the tumor suppressor *PHLPP2*, activating the PI3k/Akt pathway, promoting cancer cell growth and survival, and eventually driving tumor development and progression. Otherwise, several studies demonstrated that the expression level of miR-760 was down-regulated in other types of cancer, such as colorectal, gastric, and hepatocellular cancer^19,20,45^.

Considering miR-1973, it was up-regulated in BC patients compared to the control and benign groups. Moreover, its level was over-expressed in the patients with an early-stage BC in comparison with the controls. A study by Fomicheva et al.^30^ exhibited an elevation in miR-1973 expression levels in the MDA-MB-231 BC cell line and xenograft of mice model, which echoes our findings. Other researchers noticed an increase in miR-1973 plasma levels in endometriosis-associated ovarian cancer and Hodgkin lymphoma^27,46^. Further, the miR-1973 level was over-expressed in prostate and colon cancer tissues^26,29^. In contrast, Munari et al.^28^ reported that the miR-1973 expression levels were decreased in the tissue of clear cell papillary renal cell carcinoma patients. Of note, the mechanism of action of miR-1973 in cancer is still poorly defined, and available studies reported expression changes and correlations.

The value of miR-760 and miR-1973 expression levels as diagnostic biomarkers for other solid tumors has been assessed in previous studies^19,22,26,27^. Herein, ROC curve analysis was performed to explore the diagnostic ability of miR-760 and miR-1973 circulating levels in distinguishing BC patients from controls and BBH patients. The results displayed that miR-760 and miR-1973 have a better diagnostic impact than CA 15.3. However, the combinational analysis of miR-760 and miR-1973 with CA 15.3 enhanced the diagnostic performance of the latter. Worth mentioning, the ROC analysis demonstrates AUC values between 0.70 and 0.77, indicating moderate diagnostic performance. Although statistically significant, the clinical applicability for early detection may be limited.

While the exact role of miR-760 and miR-1973 in BC remains unclear, evidence suggests their involvement in tumor progression. MiR-760 was upregulated in CCL18-stimulated exosomes derived from MDA-MB-468 cells. Moreover, miR-760 acted as an onco-miR and targeted the *ARF6* gene, activating the PI3k/Akt signaling pathway and promoting BC cell proliferation, migration, and invasion^42^. In addition, Fomicheva et al.^30^ observed an association between the high expression level of miR-1973 and decreased apoptosis in BC xenografts. These findings align with the results of the binary logistic regression analysis, which indicated the association between the high expression level of both miRNAs and an increased risk of BC development.

To gain more insight into the mechanism of these miRNAs in BC, bioinformatics and functional analyses were performed using several platforms. GO and KEGG enrichment analyses were conducted on their target genes. GO enrichment analysis demonstrated that the target genes are involved in the positive regulation of protein kinase B (Akt) signaling, which has been previously demonstrated in BC. The active phosphorylated form of Akt promotes cell growth, proliferation, motility, and survival by stimulating several downstream effectors, such as mTOR complex 1, and activating or inactivating various transcription factors^47,48^. Other target genes are enriched in integrin-mediated signaling pathways and actin cytoskeleton organization. The activation of integrins regulates several signaling pathways that contribute to many biological processes, such as tumor metastasis and angiogenesis^49^.

Integrins-upregulation induces tumor invasion and metastasis *via* BC’s PI3k/Akt/NF-κB pathway^50^. In addition, actin cytoskeleton dynamics and organization are essential regulators for cancer progression. Integrins are vital in actin cytoskeleton reorganization and cell movement in BC, where integrin clustering induces actin polymerization at focal adhesions, which in turn contributes to changes in cell shape and polarity, aggravating the invasive behavior of cancer^51^. In our GO analysis, ubiquitin protein ligase binding was another significant pathway associated with molecular function. Ubiquitin ligases target many substrates, which are oncoproteins or tumor suppressors, and are involved in many biological processes in cancer cells. In BC, ubiquitin ligases play vital roles in BC progression including metastasis, invasion, migration, and angiogenesis^52^.

In relevance to the KEGG pathway, the neurotrophins signaling pathway was the most significant. In BC, the binding of neurotrophins to their tyrosine kinase receptors (Trk) leads to activation of tyrosine kinases, which stimulate several downstream signaling pathways, including Ras/MAPK and PI3k/Akt pathways, which are involved in cell differentiation, proliferation, migration, and invasion^53,54^. Moreover, Trouvilliez et al.^55^ revealed that the stimulation of nerve growth factor causes the binding of CD44 to the TrkA receptors, leading to BC development and metastasis.

PPI network was constructed using STRING and visualized using Cytoscape for 180 common genes. Later, PPI network was subjected to MCODE and identified the most important targets according to their high degree and MCODE score were *BRCA1*, *CDH1*, *CCND1*, and *CD44.*

Breast cancer susceptibility gene 1 (*BRCA1*) is a tumor suppressor gene and has ubiquitin ligase activity, playing an essential role in cell proliferation, cell cycle progression, and apoptosis. It was established that *BRCA1* was reduced in BC, and this repression is implicated in cancer development^56,57^. Decreased BRCA1 protein has been observed in BC, and this decrease is associated with high histological grade and short disease-free survival^58,59^.

E-cadherin gene, *CDH1*, is a tumor suppressor gene that encodes for E-cadherin protein, which plays a crucial role in cell-cell adhesion and links with catenin to form a complex that interacts with the actin cytoskeleton to maintain cell polarity and epithelial cell integrity. Loss of E-cadherin causes changes in the intercellular junction and subsequently increases cell migration, invasion, and metastasis in various cancers, including BC^60,61^. Liu et al.^62^ found that low *CDH1* expression was significantly associated with poor prognosis in patients with BC. In addition, decreased expression level of E-cadherin was associated with lymph node metastasis in patients with invasive BC^63^.

*CD44* is known to promote BC progression and metastasis. CD44 induces the re-formation of the actin cytoskeleton and facilitates cell adhesion and motility by interaction with ERM protein^64^. Loss of *CD44*s expression has been observed in malignant BC tissues as compared to benign breast tissues, providing the link between its loss and increased risk of metastasis^65,66^. Further, Huang et al.^67^ found that miR-373 and miR-520c stimulate tumor migration and invasion through direct suppression of *CD44* in MCF-7 cell line. Collectively, these findings shed light on the biological processes and molecular mechanisms of the target genes of these miRNAs in BC. However, future studies are required to confirm the clinical relevance of our findings.

Finally, the prognostic potential of the studied miRNAs among 199 patients with BC was assessed by using the KM plotter online database. On one hand, the obtained results emphasized that BC patients with high levels of miR-760 had shorter OS compared to those with a lower expression level. This is in agreement with the results of Sun et al.,^68^ who indicated that low expression of miR-760 was associated with higher OS in hepatocellular carcinoma. In contrast, downregulation of miR-760 was associated with a shorter overall and disease-free survival in gastric cancer patients^69^. On the other hand, no significant difference was noticed in OS between BC patients with high and low levels of miR-1973.

## Conclusions

The present study revealed an overexpression of circulating miR-760 and miR-1973 in BC patients, and they appear to be non-invasive diagnostic biomarkers, especially when combined with CA 15.3. In addition, the high expression levels of these miRNAs were associated with a higher risk of BC development.

### Study limitations

Some limitations should be noted in this study. First, the results of this work indicate associations instead of causal relationships between the examined parameters due to the study’s nature. Second, univariable Cox proportional hazards models and multivariable Cox regression models adjusted for clinical covariates were not performed because access to individual-level covariates is limited by the KM plotter platform. Third, false positivity is likely in the bioinformatic workflow because of the lack of filtering criteria in selecting BC-related genes. Further, being a single-center study could restrict the generalizability of the results. Finally, the relatively small sample size reduces the strength of the conclusions. Thus, future multicenter longitudinal studies with larger cohorts are necessary to establish causality and confirm the present findings. Moreover, experimental validation of miR-760 and − 1973 predicted targets would significantly strengthen the claims regarding molecular mechanisms in BC.

## Supplementary Information

Below is the link to the electronic supplementary material.

Supplementary Material 1

Supplementary Material 2

Supplementary Material 3

Supplementary Material 4

Supplementary Material 5

Supplementary Material 6

Supplementary Material 7

## Data Availability

Data is provided within the manuscript or supplementary information files.
