# Supplementary material for: Insight into the potential significance of miR-760 and miR-1973 in breast cancer: a comprehensive analysis
Source: Sci Rep. 2026 Apr 1;16:10994. doi: 10.1038/s41598-026-44175-3 (PMC13043734; doi:10.1038/s41598-026-44175-3)
Supplement: Supplementary file 1 — Supplementary Material 1 [file 41598_2026_44175_MOESM1_ESM.docx]

**Supplementary Table 1: Association of miR-760 with clinicopathological features in BC patients**

|  | **Mir-760 expression** | |  |
| --- | --- | --- | --- |
|  | **Low (n=49)** | **High (n=51)** | ***p*-value** |
| **Family history, n (%)**  **Yes**  **No** | 15 (30.6%)  34 (69.4%) | 13 (25.5%)  38 (74.5%) | 0.568 |
| **Menstruation, n (%)**  **Pre- menopause**  **Post- menopause** | 31(63.3%)  18(36.7%) | 37(72.5%)  14(27.5%) | 0.320 |
| **Histological grade, n (%)**  **I**  **II**  **III** | 4(8.2)  30 (61.2)  15 (30.6) | 2(3.9)  42 (82.4)  7 (13.7) | 0.060 |
| **Stage, n (%)**  **I**  **II**  **III**  **IV** | 10 (20.4)  16 (32.7)  21 (42.9)  2 (4.1) | 8 (15.7)  10 (19.6)  15 (29.4)  18 (35.3) | 0.002 |
| **Lymph nodes, n (%)**  **Positive**  **Negative** | 39 (79.6)  10 (20.4) | 45 (88.2)  6 (11.8) | 0.239 |
| **ER status, n (%)**  **Positive**  **Negative** | 37 (75.5)  12 (24.5) | 35 (68.6)  16 (31.4) | 0.443 |
| **PR status, n (%)**  **Positive**  **Negative** | 39 (79.6)  10 (20.4) | 36 (70.6)  15 (29.4) | 0.299 |
| **HER-2 status, n (%)**  **Positive**  **Negative** | 13 (26.5)  36 (73.5) | 18 (35.3)  33 (64.7) | 0.344 |
| **Molecular subtype, n (%)**  **Luminal A**  **Luminal B**  **HER-2 Enriched**  **Triple negative** | 24 (49)  14 (28.6)  8 (16.3)  3 (6.1) | 10 (19.6)  21 (41.2)  15 (29.4)  5 (9.8) | 0.021 |

ER: estrogen receptor; PR: progesterone receptor; HER-2: human epidermal growth factor receptor-2. Patients were divided according to the median of miR-760
