# Supplementary material for: Insight into the potential significance of miR-760 and miR-1973 in breast cancer: a comprehensive analysis
Source: Sci Rep. 2026 Apr 1;16:10994. doi: 10.1038/s41598-026-44175-3 (PMC13043734; doi:10.1038/s41598-026-44175-3)
Supplement: Supplementary file 2 — Supplementary Material 2 [file 41598_2026_44175_MOESM2_ESM.docx]

**Supplementary Table 2: Association of miR-1973 with clinicopathological features in BC patients**

|  | **Mir-1973 expression** | |  |
| --- | --- | --- | --- |
|  | **Low (n=50)** | **High (n=50)** | ***p*-value** |
| **Family history, n (%)**  **Yes**  **No** | 12 (24%)  38 (76%) | 16 (32%)  34 (68%) | 0.373 |
| **Menstruation, n (%)**  **Pre-menopause**  **Post-menopause** | 28 (56%)  22 (44%) | 40 (80%)  10 (20%) | 0.010 |
| **Histological grade, n (%)**  **I**  **II**  **III** | 4 (8)  32 (64)  14 (28) | 2 (4)  40 (80)  8 (16) | 0.203 |
| **Stage, n (%)**  **I**  **II**  **III**  **IV** | 10 (20)  10 (20)  20 (40)  10(20) | 8 (16)  16 (32)  16 (32)  10 (20) | 0.562 |
| **Lymph nodes, n (%)**  **Positive**  **Negative** | 40 (80)  10 (20) | 44 (88)  6 (12) | 0.275 |
| **ER status, n (%)**  **Positive**  **Negative** | 36 (72)  14 (28) | 36 (72)  14 (28) | 1.000 |
| **PR status, n (%)**  **Positive**  **Negative** | 41 (82)  9 (18) | 34 (68)  16 (32) | 0.106 |
| **HER-2 status, n (%)**  **Positive**  **Negative** | 13 (26)  37 (74) | 18 (36)  32 (64) | 0.280 |
| **Molecular subtype, n (%)**  **Luminal A**  **Luminal B**  **HER-2 Enriched**  **Triple negative** | 18 (36)  20 (40)  8 (16)  4 (8) | 16 (32)  15 (30)  15 (30)  4 (8) | 0.397 |

ER: estrogen receptor; PR: progesterone receptor; HER-2: human epidermal growth factor receptor-2. Patients were divided according to the median of miR-1973
